# How Does Turner Syndrome Affect Quality of Life? A Systematic Review

**DOI:** 10.3390/medicina61091643

**Published:** 2025-09-10

**Authors:** Hristina Stoynova, Radiana Staynova, Daniela Kafalova

**Affiliations:** Department of Organisation and Economics of Pharmacy, Faculty of Pharmacy, Medical University of Plovdiv, 4002 Plovdiv, Bulgaria; hristina.stoynova@mu-plovdiv.bg (H.S.); daniela.kafalova@mu-plovdiv.bg (D.K.)

**Keywords:** Turner Syndrome, monosomy X, quality of life, hormone replacement therapy, female patients, psychosocial adaptation

## Abstract

*Background and Objectives:* Turner Syndrome (ORPHA:881) is a rare genetic disorder characterized by complete or partial loss of one of the X chromosomes (monosomy X). In addition to specific somatic features and hormonal imbalances, affected individuals often face challenges related to quality of life (QoL). The aim of this review is to analyze the available scientific literature on the QoL in patients with Turner Syndrome. *Materials and Methods:* A systematic literature review was conducted using the databases Scopus, PubMed, and Web of Science, using specific key words. The search was performed from the date of inception of each database through July 2025. Only full-text articles in English assessing the QoL in individuals with Turner Syndrome were included. *Results*: The search identified 843 records, of which 12 studies met the eligibility criteria and were included in the final analysis. These studies consistently reported significant differences in the QoL between patients with Turner Syndrome and healthy controls. Factors such as hormone replacement therapy, social support, and comorbid conditions were highlighted as key determinants of QoL. *Conclusions:* Assessing the QoL in patients with Turner Syndrome is essential for understanding their overall adaptation and the need for a multidisciplinary approach to care. The findings highlight the necessity of additional long-term studies to better understand the impact of various therapeutic strategies on the QoL. The systematic review of the literature underscores the importance of a multidisciplinary approach in the care of patients with Turner Syndrome and the need for individualized therapeutic strategies aimed at improving their health-related quality of life.

## 1. Introduction

Turner Syndrome (TS, ORPHA:881) is a rare genetic disorder and one of the most common chromosomal abnormalities, affecting approximately 1 in every 2500 live-born females [1]. It is associated with multisystem involvement throughout the lifespan, thereby requiring a multidisciplinary model of care. The syndrome was first described in 1938 by the American endocrinologist Henry Turner, who reported characteristic clinical features such as short stature and primary hypergonadotropic hypogonadism [2]. TS is associated with a broad spectrum of genetic anomalies, most commonly involving the partial or complete loss of one X chromosome. It may present as monosomy X or as mosaicism involving two or more distinct cell lines. Approximately 50% of affected individuals exhibit a 45,X karyotype, while the remainder demonstrate mosaic forms, an X isochromosome, or structural anomalies involving partial or complete Y chromosome material. Importantly, in cases of mosaic karyotypes involving Y-chromosomal material, there is an approximately 20% risk of developing gonadoblastoma. Moreover, these patients do not have the potential for pregnancy, in contrast to other mosaic forms such as 46,XX/45,X. The condition is marked by a range of phenotypic manifestations affecting physical and cognitive development, with particular involvement of the endocrine, cardiovascular, reproductive, auditory, and visual systems [3].

Given this broad clinical variability, it is important to recognize that, despite individual differences, a characteristic neuropsychological profile is frequently observed in TS. This profile may negatively influence academic performance, social interactions, and emotional well-being [4]. Moreover, evidence suggests that the parental origin of the single X chromosome may affect socialization abilities, with girls retaining a maternally derived X showing greater social difficulties than those with a paternally derived X [5]. Therefore, it is crucial to evaluate how the diverse clinical manifestations of TS and their respective treatments impact health-related quality of life (HRQoL) [6]. 

The concept of HRQoL encompasses an individual’s self-reported functioning and well-being across physical, psychological, and social domains. Functional aspects include physical abilities (e.g., mobility, self-care), role performance in occupational or domestic contexts, and social interactions. In contrast, well-being reflects subjective experiences such as emotional states (e.g., happiness, sadness, and anxiety), as well as physical sensations like pain and fatigue [7]. Within this context, physical activity emerges as a relevant and modifiable factor associated with the reduced risk of both chronic somatic and mental health conditions, as well as improved overall quality of life (QoL). Preliminary evidence suggests that adolescent girls and women with TS may be less inclined to participate in or derive enjoyment from physical activity compared to their peers [8].

In addition to these broader QoL concerns, one of the most distinctive features of TS, distinguishing it from other comorbidities, is ovarian insufficiency. This results from a rapid and progressive depletion of oocytes and is observed in the majority of individuals with TS. It typically presents as absent or delayed pubertal development in adolescent girls or as infertility in women of reproductive age [2].

**Figure 1:**
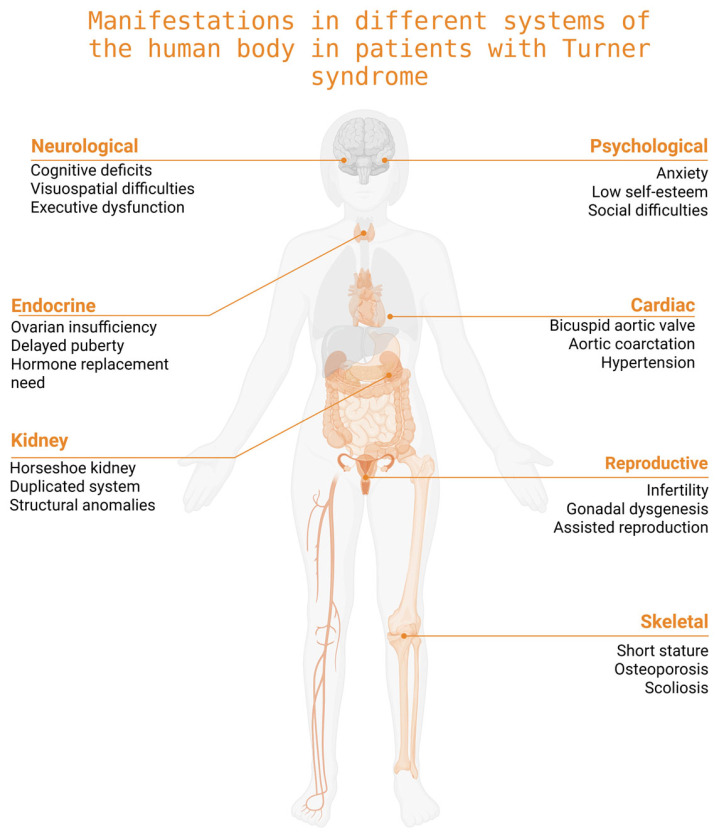
Manifestations in different systems of the human body in patients with Turner Syndrome (created with BioRender.com, accessed on 18 August 2025).

As a result, approximately 90% of individuals with TS require hormone replacement therapy to initiate and sustain pubertal development, support the maintenance of secondary sexual characteristics, and preserve bone health. Given the high risk of premature ovarian failure, early diagnosis is essential, along with the timely assessment of ovarian reserve. In cases where residual ovarian function is present, referral to a specialist in assisted reproductive technologies is recommended to discuss potential options for fertility preservation [3]. These impairments in sexual development represent a significant aspect of the clinical burden associated with TS and are among the key factors negatively impacting the overall quality of life in affected individuals (Figure 1).

Considering the complex and multisystemic nature of TS, primary care physicians play a crucial role in coordinating multidisciplinary care, as well as in the direct management of associated risk factors and complications, including infertility, cardiovascular abnormalities, and osteoporosis. However, despite the clinical importance of these interventions, there is a notable lack of controlled studies focusing on patient-centered outcomes such as morbidity, mortality, and quality of life in individuals with TS [9]. 

## 2. Materials and Methods

### 2.1. Search Strategy and Selection Criteria

A systematic literature review was conducted on the Scopus, PubMed, and Web of Science databases. The search strategy combined the terms “Turner Syndrome” OR “Monosomy X” with “Quality of Life” OR “Health-Related Quality of Life” OR HRQoL, and terms describing the population (patients, individuals, women, and girls). Full-text articles in English that assessed QoL in individuals with TS were eligible for inclusion. The search was performed from the date of inception of each database through July 2025.

**Figure 2:**
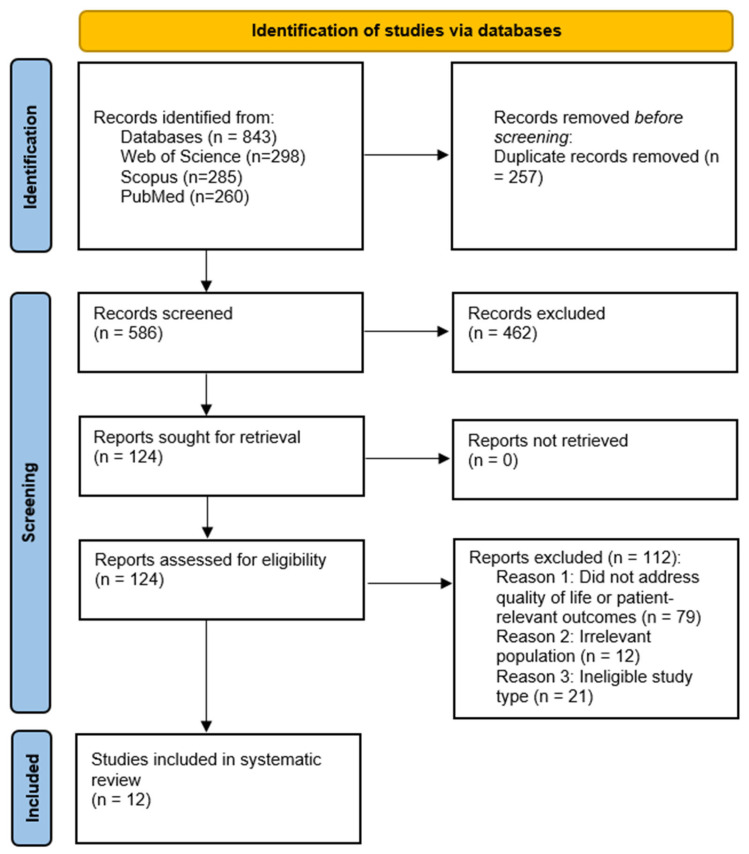
PRISMA flowchart for study selection.

The searches retrieved 843 records (Web of Science = 298; Scopus = 285; and PubMed = 260). After removing 257 duplicates, 586 unique records were screened by title and abstract, of which 462 were excluded. The full texts of the remaining 124 articles were evaluated for eligibility, resulting in 12 studies that met all inclusion criteria and were included in the review. The study-selection process followed the Preferred Reporting Items for Systematic Reviews and Meta-Analyses (PRISMA 2020) guidelines and is illustrated in PRISMA flow diagram [10] (Figure 2).

### 2.2. Eligibility Criteria

Studies were considered eligible for inclusion if they were full-text research articles written in English that specifically assessed QoL or HRQoL in individuals diagnosed with TS. The studies needed to include a direct assessment or discussion of QoL outcomes using validated instruments or well-defined qualitative measures. Articles were excluded if they were not available in full text, not written in English, or if they did not directly address QoL outcomes in the target population. The following types of publications were also excluded: systematic reviews, meta-analyses, narrative reviews, case reports, qualitative studies, editorials, commentaries, letters to the editor, and conference abstracts. A formal quality appraisal of the included studies was not performed because of the heterogeneity of study designs and outcomes and the relatively small number of eligible studies. Instead, emphasis was placed on synthesizing the available evidence to provide a comprehensive overview of QoL in TS. In addition, studies were excluded if they did not assess QoL using validated instruments or if QoL outcomes were reported only as secondary findings (e.g., studies primarily focused on body image disturbances).

The eligibility criteria were structured according to the PICOS framework (Population, Intervention, Comparison, Outcome, and Study Design) and are summarized in Table 1.

### 2.3. Data Extraction

One author (H.S.) performed the initial search in the selected electronic databases. In the next stage, duplicate records were identified and removed manually using Microsoft Excel spreadsheets. Two authors (H.S. and R.S.) independently screened the titles and abstracts of the identified articles to exclude ineligible studies. The full texts of papers that met the inclusion criteria were retrieved and assessed by one author (H.S.) and rechecked by a second author (R.S.). In case of any discrepancies, they were resolved through discussion with a third reviewer (D.K.).

The following relevant data were extracted from each publication meeting the inclusion criteria:

(1) Primary author and year of publication;

(2) Country;

(3) Study design;

(4) Sample size;

(5) Objective;

(6) Study outcomes and main results.

### 2.4. Data Synthesis

A quantitative meta-analysis or pooled effect size calculation was not feasible due to the heterogeneity of study designs, QoL instruments, and reported outcomes. Instead, a narrative synthesis of the findings was performed.

## 3. Results

A total of 843 records were retrieved from the electronic databases during the initial search, including 298 from Web of Science, 285 from Scopus, and 260 from PubMed. After the removal of 257 duplicate records, 586 records remained for screening.

Following the title and abstract screening, 462 records were excluded as irrelevant to the objectives of this review. The full texts of the remaining 124 articles were retrieved and assessed for eligibility. Of those, 112 were excluded for various reasons (as detailed in Figure 2—PRISMA figure). Ultimately, 12 studies were included in the scoping review in Table 2.

### 3.1. Characteristics of Studies Included

Of the 12 studies included in this review, the majority were observational in design, comprising cross-sectional studies *(n* = 8, 61.5%) [11,13,14,18,20,21,22], one longitudinal cohort study (*n* = 1, 7.7%) [16], and one controlled cross-sectional study (*n* = 1, 7.7%) [15]. Additionally, one randomized controlled trial (*n* = 1, 7.7%) [17], one prospective observational study (*n* = 1, 7.7%) [12], and one preliminary comparative study *(n* = 1, 7.7%) [19] were identified. Regarding the country of origin, two studies were conducted in Sweden [16,18], two in Canada [12,16], and one each in the USA [11], the Netherlands [12], Austria [14], France [15], Turkey [19], Poland [20], Malaysia [21], and Japan [22]. The studies were published between 1994 and 2023.

The objectives of the included studies varied. Several aimed to evaluate HRQoL and psychosocial functioning among adult women with TS [12,14,16,17,18,20], while others focused on children or adolescents [11,15,19]. Two studies specifically assessed the impact of growth hormone (GH) therapy on adult height and HRQoL outcomes [13,17]. A subset of studies further explored factors associated with psychological well-being, such as hearing impairment, age at diagnosis, and social integration [16,17,18,20]. Sample sizes ranged from 7 participants [19] to 178 [16]. Instruments for assessing the quality of life and related domains included the SF-36 [13,14,17], EQ-5D [12], PedsQL™ [15], Psychological General Well-Being Index [16,18], WHOQOL-BREF [21], and other validated questionnaires.

Overall, most studies reported a lower HRQoL among individuals with TS compared to controls, with particular challenges in physical functioning, social relationships, and psychological well-being. A subset of studies highlighted the role of early psychosocial support and a structured healthcare transition in mitigating these outcomes [14,15,20].

### 3.2. Quality of Life in Children and Adolescents with Turner Syndrome

The impact of TS on QoL during childhood and adolescence has been examined in several studies employing both quantitative and qualitative approaches. Overall, findings indicate substantial challenges across physical, psychosocial, and cognitive domains, with implications for health promotion and supportive care strategies (Table 3).

Thompson et al. [11] conducted a mixed-methods study combining standardized questionnaires and in-depth interviews to assess physical activity and QoL among adolescent girls with TS and their parents. Only 19% of participants met the recommended levels of moderate-to-vigorous physical activity, and both adolescents and their parents reported significantly reduced physical activity compared to controls. Barriers included fatigue, short stature, clumsiness, motor incoordination, and anxiety. The study also showed that regular exercise programs with social support are important for getting people involved and improving their well-being.

Similarly, Amedro et al. [15] performed a controlled cross-sectional study evaluating the HRQoL in children with TS compared to age-matched controls. Results demonstrated significantly lower HRQoL scores in the TS group, particularly in the domains of physical functioning, emotional well-being, and school performance, as measured by the PedsQL™ instrument. These findings highlight the early and pervasive psychosocial impact of TS and the need for proactive interventions. These results show that TS has early and widespread psychosocial impacts, and this highlights the importance of early action.

In contrast, Karakök et al. [19] assessed neurocognitive and psychosocial functioning in adolescents with TS relative to peers with a short stature and normal karyotype. Although patients with TS showed a noticeably lower cognitive performance, their self-reported quality of life, self-esteem, and coping methods were not significantly different from others. This suggests that obtaining an early diagnosis and receiving targeted, team-based care might help these young people adapt socially and emotionally, potentially reducing certain negative outcomes noted in other cohorts.

Taken together, these studies emphasize the multifaceted nature of QoL concerns in children and adolescents with TS. One of the frequently identified characteristics of the condition involves reduced physical activity levels, emotional and social difficulties, and cognitive challenges. Interventions that promote physical activity, provide psychological support, and address specific developmental needs are critical to improving long-term outcomes in this vulnerable population.

### 3.3. HRQoL in Adult Women with Turner Syndrome

HRQoL among adult women with TS has been investigated across multiple studies, revealing a complex interplay between medical comorbidities, psychosocial adaptation, and treatment history.

Van den Hoven et al. [12] examined the HRQoL and psychosocial functioning in a large cohort of adult women with TS. Using a comprehensive set of patient-reported outcome measures (EQ-5D, PSS-10, and CIS-20), the study found that women with TS reported a significantly lower HRQoL compared to the general population, with greater levels of stress and fatigue identified as key factors to a reduced well-being.

Krantz et al. [16] conducted a longitudinal cohort study in Sweden, evaluating the HRQoL over a 20-year follow-up period. This study found no significant association between prior growth hormone treatment and adult HRQoL, despite the expected gains in adult height. Instead, higher age, later age at diagnosis, and hearing impairment emerged as key determinants of a lower HRQoL. Overall, the women with TS reported HRQoL levels comparable to those of women in the general population, suggesting substantial adaptation over time.

Other studies have further elaborated on the psychosocial aspects of HRQoL. Taback and Van Vliet [17] reported similar findings in a long-term randomized controlled trial of growth hormone supplementation: no significant differences in the HRQoL were detected between GH-treated and untreated groups. Wide Boman et al. [18] identified somatic and social correlates of psychological well-being, highlighting that a lack of hormone replacement therapy in adulthood, hearing impairment, and later diagnosis were significantly associated with poorer outcomes. Finally, Jeż et al. [20] demonstrated that dissatisfaction with body height, loneliness, and feelings of being disabled were strong predictors of reduced life satisfaction in Polish women with TS.

Collectively, these findings underscore the multidimensional determinants of HRQoL in adult women with Turner Syndrome. While many women adapt well and report levels of well-being similar to the general population, specific factors—such as fatigue, perceived stress, hearing loss, and inadequate transition to adult care—consistently emerge as critical influences on quality of life. Addressing these domains through structured, multidisciplinary, and individualized care pathways remains essential for optimizing outcomes across the lifespan.

### 3.4. Role of Growth Hormone Therapy and Estrogen Replacement

The use of GH therapy and estrogen replacement has been extensively studied as part of the standard management of TS, with the primary aim of improving adult height and inducing pubertal development. However, the impact of these interventions on HRQoL remains less clear.

Joanne Rovet and Van Vliet [13] conducted a longitudinal randomized controlled trial assessing GH supplementation and psychosocial functioning in girls with TS, with follow-up until full adult height was reached. Although it led to greater growth compared to the controls without additional GH therapy, the study found no consistent global improvements in self-esteem, social functioning, or behavior across the four measurement sessions. Modest associations were observed between greater height gain and select aspects of psychosocial functioning, including fewer social problems and higher self-concept scores. However, both treated and untreated groups continued to report lower social competence. The limited improvements in psychosocial functioning among TS patients receiving GH treatment underscore the importance of exploring additional strategies to help them manage the difficulties associated with their condition.

Taback and Van Vliet found that in a follow-up of young women with Turner Syndrome, growth hormone treatment resulted in increased adult height but showed no significant association with the HRQoL. Both treated and untreated groups had HRQoL scores comparable to the general Canadian female population, and no links were observed between the final height and HRQoL measures [17].

Krantz et al. reported that in women with Turner Syndrome, no significant association was found between growth hormone treatment or final height and the HRQoL at baseline or during 20 years of follow-up. These findings suggest that height does not influence the HRQoL later in life in this population [16].

Taken together, evidence suggests that while growth hormone therapy in Turner Syndrome effectively increases adult height and supports pubertal development, its influence on the health-related quality of life and psychosocial functioning appears limited. Studies consistently report no strong association between final height and overall well-being, with only modest improvements observed in certain aspects of self-concept and social functioning. These findings underline the importance of developing additional strategies to address the psychosocial needs of individuals with Turner Syndrome beyond height augmentation alone.

### 3.5. Comorbidities in Patients with Turner Syndrome

The presence of comorbidities is characteristic of TS and has a significant impact on quality of life, functional performance, and psychosocial well-being. Studies show that a wide range of medical complications—such as cardiovascular, metabolic, orthopedic, sensory, and reproductive problems—are prevalent across the lifespan of individuals with TS.

Van den Hoven et al. [12] reported that adult women with TS frequently experience increased fatigue, stress, and a reduced HRQoL, with ontological problems, cardiovascular complications, reduced thyroid function, and orthopedic issues identified as key contributors to poorer health outcomes. Ertl. et al. [14] similarly found that obesity and hypertension were strongly associated with lower SF-36 scores in physical and social-functioning domains. In addition, Krantz et al. [16] highlighted the relevance of later diagnosis and hearing loss as independent predictors of reduced HRQoL.

Musculoskeletal and sensory comorbidities are also commonly reported. Wide Boman et al. [18] noted that the absence of hormone replacement therapy, hearing deficits, and a higher age at menarche were linked to impaired psychological well-being. Okada [22] observed high rates of body image-related anxiety, shoulder stiffness, dermatological complications, and general fatigue among adult women. In younger populations, Amedro et al. [15] documented that physical limitations were evident even in childhood, contributing to lower HRQoL scores compared to controls.

Taken together, these findings illustrate that comorbid conditions in TS are diverse and have a cumulative impact on daily functioning and perceived well-being. Comprehensive care addressing both physical and psychological needs is vital to improve lifelong outcomes.

### 3.6. Measurement Tools Used Across Studies

A variety of validated instruments were employed across studies to assess the HRQoL, psychological well-being, and related outcomes in individuals with TS. The most frequently utilized tools included generic HRQoL questionnaires and domain-specific measures (Table 4).

Several studies relied on the Short Form-36 Health Survey (SF-36) to evaluate physical and mental health components consisting of physical functioning (PF), role-physical (RP), bodily pain (BP), general health (GH), vitality (VT), social functioning (SF), role-emotional (RE), and mental health (MH) [13,14,17]. This instrument allows for comparison with normative population data and provides composite scores for physical functioning, role limitations, and emotional well-being.

The EQ-5D, a standardized measure of health status and utility, was applied in Van den Hoven et al. [12] to capture global HRQoL perceptions and to illustrate how much the disease affects people.

For children and adolescents, the Pediatric Quality of Life Inventory (PedsQL™ 4.0) was used to assess physical, emotional, social, and school functioning [15,19].

Psychological well-being was further evaluated with the Psychological General Well-Being Index (PGWB) in Krantz et al. [16] and Wide Boman et al. [18], offering a perspective on subjective distress and perceived vitality.

Yusof et al. [21] used the WHOQOL-BREF, a widely accepted instrument for assessing quality of life across physical, psychological, social, and environmental domains.

Additionally, studies employed disease-specific and complementary measures such as the Body Image Disturbances Questionnaire (BIDQ) [22], the Perceived Stress Scale (PSS-10), and the Checklist Individual Strength (CIS-20) [12]. In mixed-method designs, quantitative surveys were supplemented by semi-structured interviews to identify experiential factors related to the HRQoL and psychosocial adaptation [11].

Collectively, the diversity of tools reflects the multidimensional nature of life with TS and the necessity of integrating physical, emotional, and social assessments when evaluating outcomes in this population. 

## 4. Discussion

This scoping review gathered evidence from 12 studies examining HRQoL, psychological well-being, and social adaptation among individuals with TS. Overall, the findings highlight that, despite advances in diagnosis and treatment, TS remains associated with significant challenges across physical, psychosocial, and functional domains throughout the lifespan. While growth hormone therapy has been widely adopted and shown to improve adult height, its impact on the HRQoL appears limited. Several studies consistently reported no significant association between final stature and subjective well-being, suggesting that somatic treatment alone may be insufficient to address the broader aspects of life satisfaction and psychological adjustment in this population.

In addition to height-related concerns, TS is characterized by a complex interplay of medical and psychosocial factors that shape quality of life. Organ abnormalities, cardiovascular comorbidities, and metabolic risks are prevalent and contribute to increased morbidity and mortality [1,23]. Moreover, infertility and premature ovarian failure are frequently cited as sources of emotional distress and diminished self-esteem, underscoring the importance of early counseling and fertility preservation strategies [3,24]. It is important to note that findings across studies are not always consistent. For instance, while some investigations reported improvements in QoL following growth hormone therapy, others did not demonstrate significant benefits. These discrepancies may be explained by differences in study design, sample sizes, age at treatment initiation, duration of follow-up, and the specific QoL instruments applied. Such heterogeneity underscores the need for cautious interpretation of results and highlights the importance of standardized outcome measures in future research. In addition, specific clinical implications arise in TS mosaic cases with Y-chromosomal material. These patients face a markedly increased risk of gonadoblastoma, estimated at around 20%, and unlike other mosaic forms, they do not have reproductive potential. Such risks further compound the psychosocial burden and highlight the need for vigilant surveillance and counseling in this subgroup. Current clinical practice guidelines emphasize the necessity of a multidisciplinary approach to care that integrates endocrinological management with psychological and social support throughout childhood, adolescence, and adulthood [25].

Notably, studies included in this review also described persistent difficulties in social adaptation, including feelings of isolation, low self-confidence in peer relationships, and impaired social competence. These social difficulties may also be influenced by the parental origin of the X chromosome. A clinical report [5] described that girls with a maternally inherited X exhibited more pronounced deficits in social cognition and peer interactions compared with those with a paternally inherited X, suggesting that genetic imprinting plays a role in the psychosocial phenotype of TS. An additional consideration is the distinction between standardized HRQoL instruments and subjective self-perceptions. Validated questionnaires provide a structured and comparable assessment of quality of life but may not fully capture the nuanced lived experiences of individuals with TS. In contrast, self-reported perceptions reflect the immediate emotional and social realities of patients yet can be influenced by individual expectations and coping strategies. Integrating both objective and subjective perspectives is therefore essential for a more comprehensive understanding of QoL in TS. These observations align with the neurocognitive profile frequently described in TS, which often involves limitations in visuospatial ability, executive control, and social cognition [26]. Such cognitive and psychosocial vulnerabilities may further compromise adaptation and increase the risk of anxiety and mood disorders.

Furthermore, the variability in psychosocial outcomes observed across studies highlights the influence of contextual factors, such as family support, access to specialized care, and societal attitudes toward visible differences. It is likely that these environmental and relational factors moderate the impact of medical challenges on overall well-being. For example, adolescents and young adults with TS who report stronger family cohesion and positive healthcare experiences may demonstrate greater resilience and higher quality of life despite physical comorbidities. Conversely, inadequate social support or negative stereotyping may exacerbate psychological distress and obstruct successful adaptation.

Another important consideration is the transition from pediatric to adult healthcare services, which represents a critical period of vulnerability. Evidence suggests that gaps in coordinated care and insufficient preparation for self-management can negatively affect the continuity of treatment and psychosocial outcomes. Structured transition programs and early planning are therefore recommended to support autonomy and engagement in long-term health monitoring.

Notably, studies indicate that children and adolescents with TS often report higher quality of life scores compared to adult patients. This difference may be attributed to stronger family support, fewer comorbidities, and relatively lower expectations regarding social and professional achievement during earlier life stages. With increasing age, however, the accumulation of chronic health issues—such as cardiovascular, metabolic, and musculoskeletal conditions—along with reproductive limitations and greater societal demands, may contribute to the lower quality of life frequently reported by adult women with TS.

Taken together, these insights underscore the need for comprehensive care models that address not only somatic growth and medical surveillance, but also the emotional, cognitive, and social dimensions of living with TS. Such approaches should prioritize individualized care plans, routine psychosocial assessments, and interventions aimed at fostering self-esteem, coping skills, and social participation.

### Strengths and Limitations

This review provides a broad synthesis of studies exploring quality of life and psychosocial functioning in TS. Key advantages of the review are the inclusion of diverse age groups and both quantitative and qualitative evidence. However, limitations include the heterogeneity in study designs and measurement tools, small sample sizes in some studies, and the predominance of cross-sectional data, which makes it difficult to establish comparability or draw causal conclusions. In addition, no formal assessment of study quality or risk of bias was performed, which may further limit the strength of the conclusions. Furthermore, a quantitative meta-analysis or pooled effect size calculation was not feasible due to the heterogeneity of study designs, QoL instruments, and reported outcomes. Despite these factors, the review highlights important areas for clinical focus and further research.

## 5. Conclusions

In summary, this review demonstrates that individuals with TS face persistent challenges affecting their HRQoL, including comorbidities, body image concerns, and psychosocial difficulties. While medical treatments such as growth hormone and estrogen replacement improve physical outcomes, their impact on well-being appears limited without comprehensive psychosocial support. Early diagnosis, multidisciplinary care, and targeted interventions addressing psychological and social needs are essential to enhance long-term outcomes. Future research should prioritize longitudinal studies and the development of patient-centered strategies to improve life satisfaction in this population.

## Figures and Tables

**Table 1:** PICOS criteria for study selection.

Parameter	Description
**Population**	Individuals diagnosed with TS (any age group)
**Intervention**	Studies assessed QoL outcomes without examining the effect of a defined intervention.
**Comparison**	Studies with or without a control group (e.g., general population, other clinical conditions)
**Outcome**	Assessed QoL or HRQoL using quantitative or qualitative methods
**Study Design**	Cross-sectional studies, cohort studies, pre-post studies, interventional prospective studies, case–control studies, and other original research articles; reviews, editorials, and commentaries were excluded

**Table 2:** Characteristics of included studies.

Author, Year	Country	Study Design	Sample Size	Objective	Study Outcomes and Main Result
Thompson et al., 2020 [11]	USA	Mixed methods (cross-sectional + interviews) Qualitive + quantitative methods	*n* = 21 adolescent girls with TS and 21 parents	To examine physical activity levels and QoL in adolescents with TS and explore TS-specific factors affecting physical activity.	Only 19% met activity guidelines. The Patient-Reported Outcomes Measurement Information System (PROMIS) indicated reduced physical activity and impaired peer relations (as reported by parents). Fatigue and psychosocial barriers (short stature, anxiety, and transport issues) limited activity. Structured routines and social support improved engagement.
Van den Hoven AT et al., 2020 [12]	Netherlands	Prospective observational study	*n* = 177 women with TS	To evaluate HRQoL and psychosocial functioning in adult women with TS as part of a value-based healthcare approach.	Women with TS reported significantly lower HR-QoL (EQ-5D), higher stress (PSS-10), and increased fatigue (CIS-20) compared to controls. Fatigue, stress, diabetes, and orthopedic issues were linked to poorer QoL.
Joanne F. Rovet et al., 2019 [13]	Canada	Cross-sectional, retrospective	*n* = 131 women with TS (70 on growth hormone (GH) and 61 without)	To assess the impact of GH therapy on adult height and QoL in women with TS.	Women with GH treatment were significantly taller and had higher QoL scores (measured via SF-36) than untreated women. Mental health scores were higher in GH-treated group. GH therapy showed long-term benefits beyond height increase.
Ertl. et al., 2018 [14]	Austria	Cross-sectional survey study	*n* = 39 women with TS	To assess the health status, QoL, and satisfaction with medical care among adult women with TS.	QoL was lower in TS women compared to the general population (SF-36). Poorer health (e.g., hearing loss, obesity, and hypertension) correlated with lower QoL. Many participants reported discontinuity of care after childhood, with insufficient transition from pediatric to adult healthcare. Women who received structured follow-up as adults reported better QoL and higher satisfaction with care.
Amedro et al., 2019 [15]	France	Controlled cross-sectional study	*n* = 16 girls with TS and 64 female controls	To evaluate HRQoL in children with TS compared to healthy controls.	Girls with TS showed significantly lower HRQoL scores (measured by PedsQL™ 4.0) than controls, particularly in physical, emotional, and school functioning. These findings highlight the psychosocial and functional impact of TS even in childhood, underlining the need for early support and interventions.
Krandz et al., 2019 [16]	Sweden	Longitudinal cohort (20-year follow-up)	*n* = 178 women with TS	To assess HRQoL in adult women with TS, with a focus on the impact of previous growth hormone treatment and comorbidities and to compare their HRQoL to that of women from the general population.	HRQoL was assessed using validated Swedish versions of the Psychological General Well-Being Index (PGWB) and the Nottingham Health Profile (NHP). Women with TS reported overall HRQoL comparable to that of the general population. GH treatment during childhood was not significantly associated with improved HRQoL, despite resulting in an average height gain of 5.7 cm. Lower HRQoL was significantly associated with older age, later age at diagnosis, and hearing impairment.
Taback & Van Vliet, 2011 [17]	Canada	Randomized controlled trial with long-term follow-up	*n* = 34 women with TS	To investigate whether long-term GH treatment in individuals with TS influences HRQoL in young adulthood—either positively, due to increased height or treatment perception, or negatively, due to prolonged medicalization through years of injections.	HRQoL was assessed using the SF-36 questionnaire. No significant differences were found between GH-treated and untreated groups across all domains. Despite increased adult height from GH therapy, there was no measurable benefit in HRQoL. Results suggest that height gain alone may not influence overall well-being in young adults with TS.
Wide Boman et al., 2004 [18]	Sweden	Cross-sectional observational study	*n* = 63 adult women with TS (mean age 31.5 years)	To investigate how somatic factors and social experiences, including the role of hormone replacement therapy (HRT), are associated with psychological well-being in adult women with TS.	Psychological well-being was assessed using the Psychological General Well-Being (PGWB) Index, complemented by clinical examination data and medical records. Lower well-being was associated with absence of sex hormone therapy in adulthood, hearing impairment, later age at diagnosis and menarche (or induced bleeding), older age, and reported academic difficulties. Age at diagnosis and school-related challenges together accounted for 25% of the variance in well-being. The findings underscore the relevance of these factors in the clinical care of adult women with TS.
Karakök et al., 2021 [19]	Turkey	Preliminary study	*n* = 7 adolescent girls with TS (TS), *n* = 7 with short stature (SS) and normal karyotype	To evaluate and compare the neurocognitive and psychosocial profiles of adolescents with TS and peers with short stature and normal karyotype.	No significant differences were observed between the two groups in terms of psychiatric diagnoses, social cognition skills, quality of life, self-esteem, and coping strategies. However, adolescents with TS had significantly lower scores in cognitive functioning (working memory, processing speed, and total IQ) than those with SS. Anxiety and conduct problems were more prominent in the SS group. Findings support early neurocognitive assessment and psychosocial support in TS.
Jeż et al., 2018 [20]	Poland	Cross-sectional study	*n* = 176 adult women with TS	To assess the impact of TS and its biological and psychosocial consequences on quality of life, with a focus on life satisfaction.	Life satisfaction was assessed through a standardized questionnaire and logistic regression. The main independent predictors of lower life satisfaction were dissatisfaction with short stature, loneliness, and the feeling of being disabled. Other relevant factors included social stigmatization, family attitudes, and negative self-perception. Findings emphasize the importance of psychosocial support in managing TS.
Yusof et al., 2023 [21]	Malaysia	Cross-sectional study	*n* = 24 TS patients and *n* = 60 controls	To assess the QoL and body image disturbances in adult patients with TS compared to age-matched healthy controls at a tertiary hospital in Kuala Lumpur, using the World Health Organization Quality of Life (WHOQOL-BREF) Questionnaire and the Body Image Disturbances Questionnaire (BIDQ).	TS patients had comparable overall QoL to controls but significantly lower scores in the social relationship domain. Body image concerns were significantly associated with impairment in social and occupational functioning. The main concern among TS patients was related to short stature and low self-esteem.
Okada, 1994 [22]	Japan	Cross-sectional comparative study	*n* = 20 women with TS; *n* = 22 GH-deficient women	To evaluate QoL in adult women with TS in comparison with GH-deficient women, based on aspects such as education, employment, income, social life, physical complaints, and emotional well-being.	Turner women had significantly higher university enrollment than the general population (68% vs. 38.2%), similar employment levels to controls, but experienced more body image and marriage-related anxiety. Both groups reported psychosomatic symptoms such as shoulder stiffness and fatigue.

**Table 3:** Comparison of quality-of-life dimensions in children/adolescents vs. adult women with TS.

Parameter	Children and Adolescents	Adult Women
**Physical Activity**	Significantly lower than peers; common barriers include fatigue, short stature, and motor coordination difficulties [11,15]	Reduced stamina, increased fatigue; difficulty engaging in daily activities [12,16]
**Emotional Health**	Anxiety, low self-esteem, and emotional lability [11,15,19]	Increased stress, risk of depression, and feelings of isolation [12,18,20]
**Social Integration**	Difficulties in forming friendships; sense of being “different” [11,15]	Loneliness, low self-confidence, and challenges in intimate relationships [18,20,21]
**Educational and Cognitive Functioning**	Learning difficulties—particularly in mathematics and visuospatial reasoning [19]	Executive function deficits; frequently undiagnosed cognitive impairments [16,18]
**Impact of Therapy (GH/HRT)**	GH improves height but has limited impact on QoL; early psychological support is crucial [13,17]	HRT improves physical outcomes; lack of therapy associated with reduced psychological well-being [18]
**Psychosocial Support**	Better QoL observed in presence of strong family and social support [11,15,19]	Structured care and coordinated transition to adult services enhance overall QoL [14,20]

**Table 4:** Measurement instruments used to assess HRQoL in individuals with TS.

Instrument	Age Group	Domains	Studies Using the Instrument
**SF-36 Health Survey**	Adults	Physical functioning, role–physical, bodily pain, general health, vitality, social functioning, role–emotional, and mental health	Rovet et al., 2019 [13]; Ertl. et al., 2018 [14]; Taback & Van Vliet, 2011 [17]
**EQ-5D**	Adults	Mobility, self-care, usual activities, pain/discomfort, and anxiety/depression	Van den Hoven et al., 2020 [12]
**Pediatric Quality of Life Inventory (PedsQL™ 4.0)**	Children and adolescents	Physical, emotional, social, and school functioning	Amedro et al., 2017 [15]
**Psychological General Well-Being Index (PGWB)**	Adults	Anxiety, depression, well-being, self-control, vitality, and general health	Krantz et al., 2019 [16]; Wide Boman et al., 2004 [18]
**WHOQOL-BREF**	Adults	Physical health, psychological, social relationships, and environment	Yusof et al., 2023 [21]
**Body Image Disturbances Questionnaire (BIDQ)**	Adults	Body image perception, associated social and occupational impairments	Yusof et al., 2023 [21]
**Perceived Stress Scale (PSS-10)**	Adults	Perceived stress levels	Van den Hoven et al., 2020 [12]
**Checklist Individual Strength (CIS-20)**	Adults	Fatigue, concentration, motivation, and physical activity	Van den Hoven et al., 2020 [12]

## Abbreviations

The following abbreviations are used in this manuscript:

TS	Turner Syndrome
QoL	Quality of Life
HRQoL	Health-Related Quality of Life
GH	Growth Hormone
HRT	Hormone Replacement Therapy
PROMIS	Patient-Reported Outcomes Measurement Information System
PedsQL™	Pediatric Quality of Life Inventory
PGWB	Psychological General Well-Being Index
WHOQOL-BREF	World Health Organization Quality of Life–BREF
BIDQ	Body Image Disturbances Questionnaire
PSS-10	Perceived Stress Scale (10-item version)
CIS-20	Checklist Individual Strength (20-item version)
PRISMA	Preferred Reporting Items for Systematic Reviews and Meta-Analyses

## References

[1] Yoon S.H., Kim G.Y., Choi G.T., Do J.T. (2023). Organ Abnormalities Caused by Turner Syndrome. Cells.

[2] Clemente E.G., Penukonda S.K., Doan T., Sullivan B., Kanungo S. (2022). Turner Syndrome. Endocrines.

[3] Porcu E., Cipriani L., Damiano G. (2023). Reproductive Health in Turner’s Syndrome: From Puberty to Pregnancy. Front. Endocrinol..

[4] Hutaff-Lee C., Bennett E., Howell S., Tartaglia N. (2019). Clinical Developmental, Neuropsychological, and Social-Emotional Features of Turner Syndrome. Am. J. Med. Genet. C Semin. Med. Genet..

[5] Donnelly S.L., Wolpert C.M., Menold M.M., Bass M.P., Gilbert J.R., Cuccaro M.L., Delong G.R., Pericak-Vance M.A. (2000). Female with autistic disorder and monosomy X (Turner syndrome): Parent-of-origin effect of the X chromosome. Am. J. Med. Genet..

[6] Gawlik A., Kaczor B., Kaminska H., Zachurzok-Buczynska A., Gawlik T., Malecka-Tendera E. (2012). Quality of medical follow-up of young women with Turner syndrome treated in one clinical center. Horm. Res. Paediatr..

[7] Wilson I.B., Cleary P.D. (1995). Linking Clinical Variables with Health-Related Quality of Life. JAMA.

[8] Landry B.W., Ratterman N.L., Nielson K.A., Payne J.B., Ramsey R.R. (2019). A Mixed Methods Study of Physical Activity and Quality of Life in Girls with Turner Syndrome. Am. J. Med. Genet. C Semin. Med. Genet..

[9] Morgan T. (2007). Turner Syndrome: Diagnosis and Management. Am. Fam. Physician.

[10] Page M.J., McKenzie J.E., Bossuyt P.M., Boutron I., Hoffmann T.C., Mulrow C.D., Shamseer L., Tetzlaff J.M., Akl E.A., Brennan S.E. (2021). The PRISMA 2020 Statement: An Updated Guideline for Reporting Systematic Reviews. Int. J. Surg..

[11] Thompson T., Zieba B., Howell S., Karakash W., Davis S. (2020). A Mixed Methods Study of Physical Activity and Quality of Life in Adolescents with Turner Syndrome. Am. J. Med. Genet. A.

[12] Van den Hoven A.T., Bons L.R., Dykgraaf R.H.M., Dessens A.B., Pastoor H., de Graaff L.C.G., Metselaar M.R., Kneppers-Swets A., Kardys I., Mijnarends H. (2020). A Value-Based Healthcare Approach: Health-Related Quality of Life and Psychosocial Functioning in Women with Turner Syndrome. Clin. Endocrinol..

[13] Rovet J.F., Van Vliet G. (2019). Growth Hormone Supplementation and Psychosocial Functioning to Adult Height in Turner Syndrome: A Questionnaire Study of Participants in the Canadian Randomized Trial. Front. Endocrinol..

[14] Ertl D.A., Gleiss A., Schubert K., Culen C., Hauck P., Ott J., Gessl A., Haeusler G. (2018). Health Status, Quality of Life and Medical Care in Adult Women with Turner Syndrome. Endocr. Connect..

[15] Amedro P., Tahhan N., Bertet H., Jeandel C., Guillaumont S., Mura T., Picot M.C. (2017). Health-Related Quality of Life among Children with Turner Syndrome: Controlled Cross-Sectional Study. J. Pediatr. Endocrinol. Metab..

[16] Krantz E., Landin-Wilhelmsen K., Trimpou P., Bryman I., Wide U. (2019). Health-Related Quality of Life in Turner Syndrome and the Influence of Growth Hormone Therapy: A 20-Year Follow-Up. J. Clin. Endocrinol. Metab..

[17] Taback S.P., Van Vliet G. (2011). Health-Related Quality of Life of Young Adults with Turner Syndrome Following a Long-Term Randomized Controlled Trial of Recombinant Human Growth Hormone. BMC Pediatr..

[18] Boman U.W., Bryman I., Möller A. (2004). Psychological Well-Being in Women with Turner Syndrome: Somatic and Social Correlates. J. Psychosom. Obstet. Gynaecol..

[19] Karakök B., Akdemir D., Yalçın S., Özusta H.S., Utine G.E., Doğan Ö., Şimşek Kiper P.O., Demir G.U. (2021). Psychometric and Psychosocial Evaluation of Adolescents with Turner Syndrome in a Multidisciplinary Approach: A Preliminary Study. J. Curr. Pediatr..

[20] Jeż W., Tobiasz-Adamczyk B., Brzyski P., Majkowicz M., Pankiewicz P., Irzyniec T.J. (2018). Social and Medical Determinants of Quality of Life and Life Satisfaction in Women with Turner Syndrome. Adv. Clin. Exp. Med..

[21] Yusof A.A.B., Chii M.L.S., Yusoff N.I.M., Kama R.N.I.F.R.M., Raj J.R., Ghani N.A.A., Ali A., Hong Soo Syn J., Shah S.A., Ishak N.A. (2023). The Quality of Life and Body Image Disturbances of Turner Syndrome Patients in Malaysia: A Cross-Sectional Study. BMC Women’s Health.

[22] Okada Y. (1994). The Quality of Life of Turner Women in Comparison with Grown-Up GH-Deficient Women. Endocr. J..

[23] Krzyścin M., Sowińska-Przepiera E., Gruca-Stryjak K., Soszka-Przepiera E., Syrenicz I., Przepiera A., Bumbulienė Z., Syrenicz A. (2024). Are Young People with Turner Syndrome Who Have Undergone Treatment with Growth and Sex Hormones at Higher Risk of Metabolic Syndrome and Its Complications?. Biomedicines.

[24] Viuff M., Gravholt C.H. (2022). Turner Syndrome and Fertility. Ann. Endocrinol..

[25] Gravholt C.H., Andersen N.H., Christin-Maitre S., Davis S.M., Duijnhouwer A., Gawlik A., Maciel-Guerra A.T., Gutmark-Little I., Fleischer K., Hong D. (2024). Clinical Practice Guidelines for the Care of Girls and Women with Turner Syndrome. Eur. J. Endocrinol..

[26] Kesler S.R. (2007). Turner Syndrome. Child Adolesc. Psychiatr. Clin. N. Am..

